# Genetic tropicalisation following a marine heatwave

**DOI:** 10.1038/s41598-020-69665-w

**Published:** 2020-07-29

**Authors:** Melinda A. Coleman, Antoine J. P. Minne, Sofie Vranken, Thomas Wernberg

**Affiliations:** 1 New South Wales Fisheries, National Marine Science Centre, 2 Bay Drive, Coffs Harbour, NSW 2450 Australia; 2 Southern Cross University, National Marine Science Centre, 2 Bay Drive, Coffs Harbour, NSW 2450 Australia; 3 Oceans Institute and School of Biological Sciences, University of Western Australia, 35 Stirling Highway, Crawley, WA 6009 Australia; 4 Department of Science and Environment, Roskilde University, 4000 Roskilde, Denmark

**Keywords:** Climate-change ecology, Marine biology

## Abstract

Extreme events are increasing globally with devastating ecological consequences, but the impacts on underlying genetic diversity and structure are often cryptic and poorly understood, hindering assessment of adaptive capacity and ecosystem vulnerability to future change. Using very rare “before” data we empirically demonstrate that an extreme marine heatwave caused a significant poleward shift in genetic clusters of kelp forests whereby alleles characteristic of cool water were replaced by those that predominated in warm water across 200 km of coastline. This “genetic tropicalisation” was facilitated by significant mortality of kelp and other co-occurring seaweeds within the footprint of the heatwave that opened space for rapid local proliferation of surviving kelp genotypes or dispersal and recruitment of spores from warmer waters. Genetic diversity declined and inbreeding increased in the newly tropicalised site, but these metrics were relative stable elsewhere within the footprint of the heatwave. Thus, extreme events such as marine heatwaves not only lead to significant mortality and population loss but can also drive significant genetic change in natural populations.

## Introduction

Climate change is increasing the intensity and frequency of extreme events^1,2^ with significant impacts to species and ecosystems on both evolutionary and contemporary time scales^3^. Because extreme events, by definition, exceed normal environmental conditions, they drive significant mortality, range shifts and the transition to novel ecosystem states^4–6^. However, in contrast to the ecological impacts of extreme climatic events which are often obvious and well documented, their impact on underlying patterns of genetic diversity and structure and the implications for adaptability to future change is obscure and often unknown^3,7–10^, particularly for marine systems.

**Figure 1:**
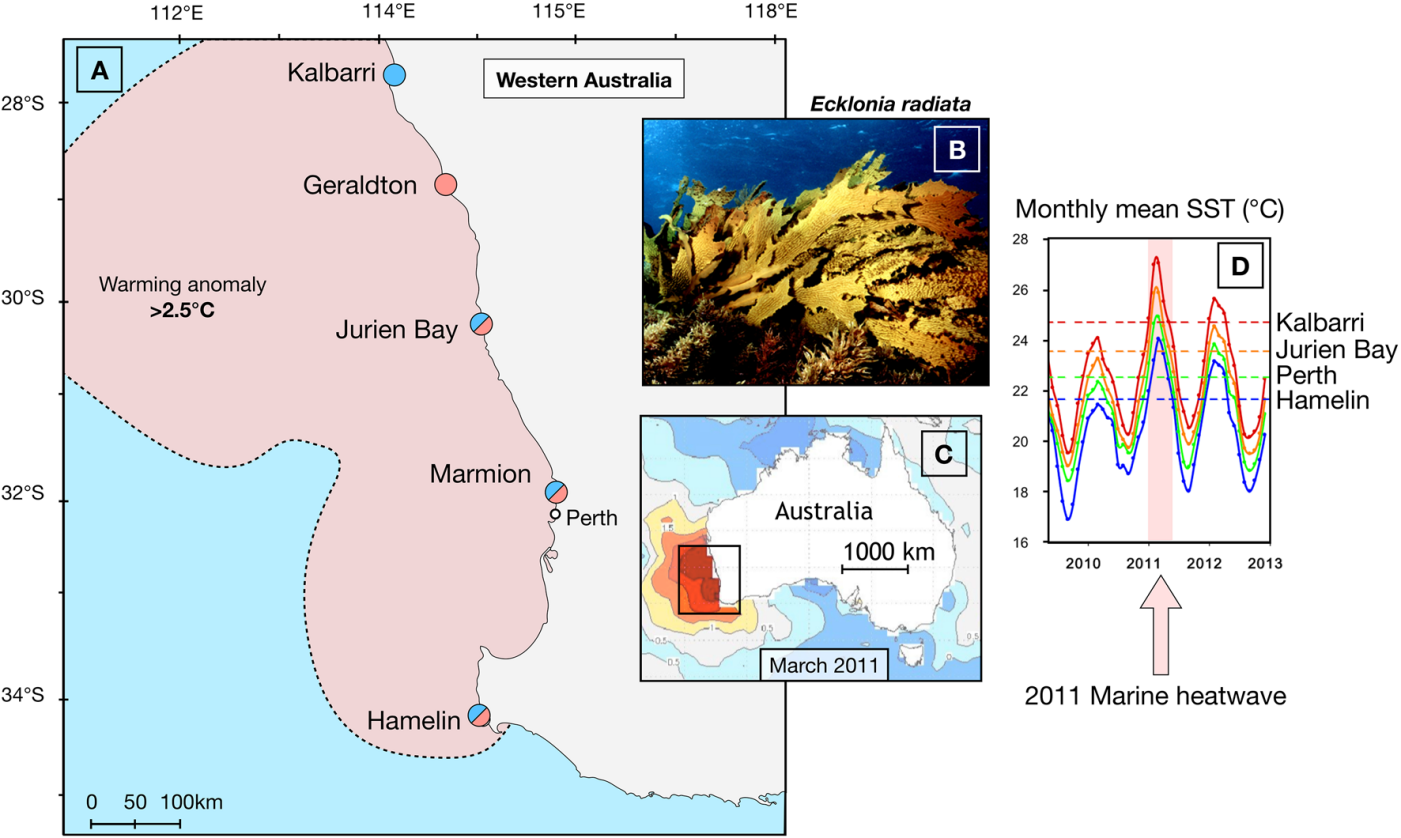
Sampling locations with footprint of the marine heatwave (red) and surface temperature anomaly reported from March 2011 compared to 1971–2000 surface temperature records^24^ (**A**), photo of *Ecklonia radiata* (**B**) and details of the regional evolution of the temperature anomaly (**C**) and at each of our study sites (**D**^10^.

Marine heatwaves are extreme events defined as discrete periods of anomalously warm-water that exceed historical norms of ocean temperature^11^ and are superimposed on a background of ocean warming. Marine heatwaves are increasing in frequency and duration globally^12^ with significant consequences for coastal marine species, communities and the ecosystem services they provide^13–17^. While we have an emerging understanding of how ocean warming may affect the genetics of marine species e.g.^18,19^, there are few empirical studies demonstrating the genetic impact of extreme marine heatwaves (but see^20,21^. One of the most severe marine heatwaves ever recorded impacted ~ 2,000 km of coast off Western Australia in 2011^22^, where sea temperatures soared up to 5.5 °C above normal for several weeks Fig. 1^23,24^. The heatwave precipitated widespread and significant local extinction and range contraction of entire marine communities^6,24,25^, shifts in ecological structure^6,26^ and impacted fisheries^27^ significantly compromising the vast ecosystem goods and services along this coastline^28^.

**Figure 2:**
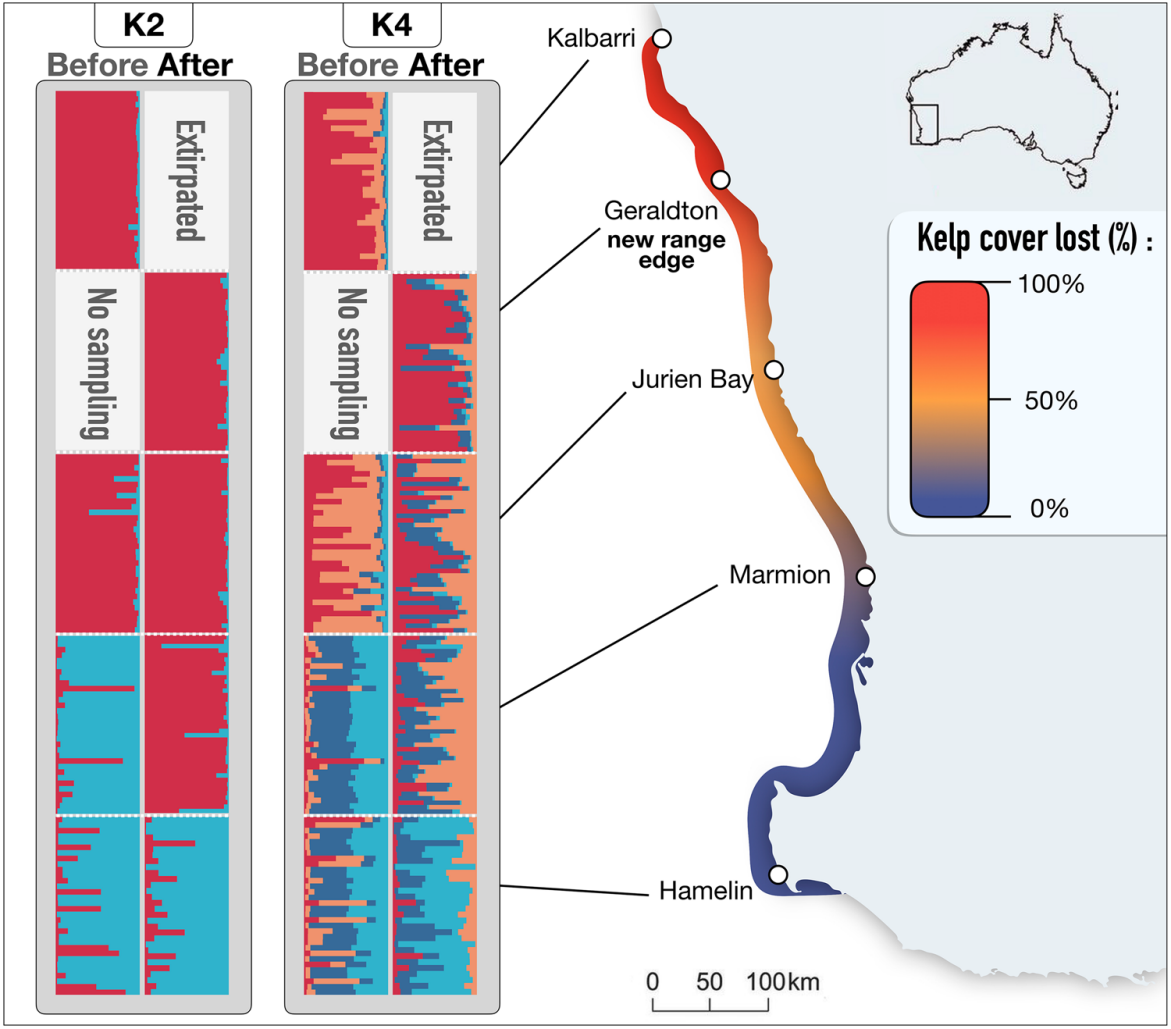
Population genetic structure inferred by STRUCTURE (v 2.3.4; https://web.stanford.edu/group/pritchardlab/structure) analysis for the K = 2 genetic clusters before and after the heatwave. K = 4 is also shown to better visualise admixture. Each horizontal line represents an individual coloured according to that individual’s membership assigned to each cluster. The gradient coloured line represents the kelp cover lost^6^.

Marine forests, comprised of foundation species of kelps and seaweeds that underpin biodiversity throughout temperate regions^28–30^, have been heavily impacted by marine heatwaves globally^13–17^ and were most heavily impacted by the 2011 Western Australian heatwave^6^. Vast underwater forests of the common kelp (*Ecklonia radiata*, hereafter referred to as *Ecklonia*,Fig. 1) were impacted by the heatwave with direct loss of kelp varying from complete local extirpation at its low latitude (warm) range edge (Kalbarri), 25–50% loss at mid latitudes and little loss at higher latitudes (Fig. 2,^10^. Subsequently, ecological communities at low and mid latitude reefs became “tropicalised”^24^ whereby the occurrence of warm-affinity species increased, shifting the entire biotic community (species compositions) to more closely resemble tropical latitudes and transforming them into novel ecosystem configurations^6^ following the heatwave. Although there is some suggestion that low genetic diversity of *Ecklonia* populations prior to the heatwave may have mediated its ecological impact^10^, the consequence of the heatwave for subsequent genetic diversity and structure of *Ecklonia* forests is currently unknown. Given that co-occurring kelp species suffered cryptic loss of genetic diversity and potential directional selection following the heatwave^20^ it is possible that *Ecklonia* populations were similarly affected. Unravelling whether the heatwave caused genetic changes in *Ecklonia* forests will help predict vulnerability to future warming and extreme events.

Because extreme events are largely unpredictable in time and space, robust empirical assessment of their impacts are often hindered by a lack of “before data”^3^. We were fortunate to have genetic samples (DNA) of *Ecklonia* forests prior to the heatwave, allowing us to provide a rare empirical demonstration of the impacts of an extreme marine heatwave on subsequent genetic properties of kelp forests. We predicted that the heatwave would precipitate loss of genetic diversity given the large mortality of *Ecklonia* at many sites and a poleward shift in warm-affinity alleles driven by an increased flow of prevailing currents during the reproductive period of *Ecklonia* immediately following the heatwave^31^.

## Materials and methods

### Marine heatwave and study species

In the Austral summer of 2010/11 the west coast of Australia experienced an unprecedented marine heatwave (MHW) with temperature anomalies exceeding anything seen in at least 140 years of recorded history Fig. 1^24^. The MHW engulfed ~ 2,000 km coastline and on the southwest coast, where this study was focused, the coastal region experienced temperature anomalies of 2.5–5.5 °C above the long term maxima (dotted lines; Fig. 1) for more than 10 weeks Fig. 1 ^23,24^. The prevailing poleward current (Leeuwin current) was also stronger during the autumn reproductive period of *Ecklonia*^31^ immediately following the heatwave.

*Ecklonia radiata* is the dominant kelp throughout Australasia^30^ where it underpins immense biodiversity^32,33^. It is a perennial species with an alternation of generations life cycle with macroscopic sporophytes (sampled here) alternating with microscopic gametophytes^30^. *E. radiata* does not reproduce asexually, except for the distinctive *E. brevipes* morphotype in Hamelin Bay^34^ which was not sampled here. Samples of *E. radiata* sporophytes for genetics were collected across 800 km of coastline that experienced the highest thermal anomalies during the heatwave (up to 5.5 °C at Jurien Bay, Fig. 1)^10,24^. Samples were collected both before (2006)^35^ and after (2018) the 2011 heatwave from Kalbarri (old range edge, before only), Geraldton (new range edge, after only), Jurien Bay, Marmion and Hamelin. There were no large thermal anomalies in the intervening years except a mildly cooler period from 2015 to 2017. We recognise that our sampling represents only 2 points in time and without more temporal replication within before and after periods we cannot definitively attribute any genetic change to the heatwave. However, we believe that the rarity of “before” genetic data in studies that seek to examine impacts of unpredictable extreme events warrants this sampling design (see^8,9,36,37^ for examples of similar designs with 2 time points for genetic data). Moreover, given that temporal replication of genetic data in any study is rare, our data is a critical first step in assessing genetic impacts of climatic change on natural systems. Both before and after the heatwave, all samples were haphazardly collected (but at least 1 m apart) on scuba or snorkel between two and ten meters depth in an area approximately 20 × 20 m^32^. Collected samples (*n* = 30–40 kelp plants per site) were free from epiphytes and were preserved in silica gel desiccant (2006) or snap frozen in liquid nitrogen (2018).

### Microsatellite genotyping and analysis

DNA from 2006 was extracted as in^35,38,39^ and in 2018 using Qiagen DNeasy plant kits and cleaned with Qiagen Powerplant Pro kits using a modified manufacturer protocol. Specifically, raw material was soaked in lysis buffer for 24–48 h at 65 °C to aid lysis and DNA was washed on the spin filter 3 times. DNA quality and quantity were checked using picogreen and gel electrophoresis (2006) or on a Qubit and Nanodrop the run on a LabChip nucleic acid analyser (Perkin Elmer) for quality control. Even DNA samples from 2006 (stored in sealed 96 well plates in a − 20 freezer) that had a lower concentration of DNA amplified well. All DNA samples were genotyped at the same time (in 2019) using *n* = 8 new polymorphic microsatellite markers developed for *E. cava*^40^ and *E. radicosa*^41^ and tested on *E. radiata* (supplementary Table S1). Microsatellites were chosen because the DNA from 2006 was of lower quantity and not suitable for RADSeq approaches that require a much larger quantity and exemplary quality of DNA. PCRs comprised a total reaction volume of 10 μl comprising 0.5 μl of template DNA (with 1:80 dilutions for most samples), 5 μl of Taq polymerase (Invitrogen Platinum II Hot Start 2 U/μl supplied by Thermo Scientific), 0.2 μl of each primer (10 μM) and 4.1 μl ddH2O. PCRs were run on an Eppendorf Thermal Cycler (Mastercycler nexus) programmed for a 2-step protocol composed of an initial denaturation at 94 °C for 2 min followed by 30 cycles each with denaturing at 98 °C for 5 s, annealing/extension at 60 °C for 15 s. Recalcitrant amplifications were repeated using MyTaq Hot Start Taq polymerase (reaction volume of 10 μl comprising 1 μl of template DNA, 5 μl of Taq polymerase (MyTaq Hot Start supplied by Bioline), 0.4 μl of each primer (10 μM) and 3.2 μl ddH_2_O). PCRs conditions were composed of an initial denaturation at 95 °C for 1 min followed by 30 cycles each with denaturing at 95 °C for 15 s, annealing at 59 °C for 15 s and extension at 72 °C for 10 s. Fragment sizes were genotyped by capillary separation at the Australian Genome Research Facility using one individual with known genotype for each loci to avoid reading errors on all sequenced plates.

Allele sizes were manually scored using the microsatellite plugin on the Geneious Prime software (v11.1). Ambiguous loci were classed as missing data. Prior to conducting statistical analyses, we checked for genotyping errors (null alleles, stutter, allele drop out and typographical errors) using MICRO-CHECKER^42^. No evidence of null alleles or scoring errors due to stuttering or large allele dropout was found in the whole data set. Overall, no more than 4% of missing data was allowed within a year with a maximum of 10% of missing data for one population across loci and 8.75% for one locus across populations. We tested for linkage disequilibrium between pairs of loci of the populations in ARLEQUIN 3.5^43^ with a permutation procedure of 10,000 iterations. We found no consistent linkage disequilibrium between loci across populations, instead the standardised index of association (implemented in the package *poppr* using 999 permutations^44^ revealed significant linkage between markers only in Geraldton after the MHW (P ≤ 0.01) which may be a result of the significant population declines in this site, however, no data was available from prior to the MHW. Departure from panmixia was tested at each locus for each population using the function *hw.test* implemented in the package *Pegas* with the conventional Chi-square test. Significance of the deviation from Hardy–Weinberg equilibrium was addressed by a Markov Chain (MC) algorithm in ARLEQUIN (exact test by Guo and Thompson^45^ tested locus-by-locus (MC: 1,106 steps; dememorization: 1,105 steps) but departure was never found for more than one locus in each population. The total number of alleles or allelic richness (Na) per locus, the observed (Ho) and expected (He) heterozygosity and the number of private alleles (unique to a particular site) were estimated for each population before and after the MHW using GENALEX 6.5^46^ and GENETIX 4.05^47^ also used to estimate pairwise Weir and Cockerham’s *F*_ST_. MLH (multi-locus heterozygosity or the mean number of heterozygous loci per individual) was calculated using the R package *InbreedR* at each site before and after the heatwave as this metric has previously been shown to be a sensitive metric of thermal change^48^. *F*_ST_ and *F*_IS_ were computed per population in FSTAT 3.2^49^. Significance of *F*_IS_ values were estimated by running 800 permutations of alleles among individuals for each population and a 5% indicative adjusted nominal level of P = 0.00125. Patterns of genetic structure were inferred by the Bayesian clustering method implemented in STRUCTURE v2.3.4 (https://web.stanford.edu/group/pritchardlab/structure) allowing admixture and independent allele frequencies enabling individuals to be assigned to different genetic groups if admixed^50^. This method is based on a Markov chain Monte Carlo (MCMC) approach to optimise genotypic equilibrium (linkage equilibrium and HWE) within each cluster. For a K (likely number of genetic clusters) ranging from 1 to 4, with no geographic prior, 10 independent runs were conducted with a burn-in period of 100,000 steps and 500,000 MCMC replications. Both mean log likelihood L(K) and ΔK were used to estimate the likely number of clusters and obtained on Structure Harvester^51^. Final bar plots were generated with the POPHELPER Structure Web App^52^ with the alignment of the assignment scores obtained over the ten runs for each K. Discriminant analyses of principal components (DAPCs**)** were computed using the R package *adegenet*^53^. This technique is used to maximise the inter-group component of variation of a principal component analysis and visually explore major genetic patterns among populations before and after the MHW. We retained 25 principal components out of the 69 (chosen as the amount of retained principal components for which the % cumulative variance slows as well as the spline interpolation from the alpha score optimisation implemented on *adegenet*) and 7 linear discriminants. The Bayesian informative criterion (BIC) was minimal for K = 8 which corresponds to the number of sampled populations when differentiating before from after. For every DAPC, loci contributions have allowed us to better identify the loci and alleles driving the observed changes in allelic frequencies between populations. Global analysis of molecular variance (AMOVA) weighted over loci was performed in ARLEQUIN using 1,000 permutations to statistically assess the differences amongst clusters. Given our unbalanced design with extirpation of kelp populations at Kalbarri and genetic change largely attributable to one site, we chose to do AMOVAs separately on data before and after the heatwave. Locus-by-locus outputs were investigated to look for main loci contributions. Pairwise *F*_ST_ tests were used to examine site differences before and after the heatwave.

Detections of recent mass mortality due to drastic effective reductions in response to extreme events such as MHW can be inferred by a heterozygosity excess compared to the expected heterozygosity based on the observed number of alleles^54^. Conversely, heterozygote deficiency is a sign of expansion. Comparisons between expected heterozygosity under HWE and the expected heterozygosity computed from the actual observed number of alleles under the assumption of mutation-drift equilibrium were performed using the two-phase model (TPM) on the BOTTLENECK v1.2.02 software^55^ (https://www1.montpellier.inra.fr/CBGP/software/Bottleneck/bottleneck) recommended for microsatellite data^54^ and was run for 1,000 iterations with a variance among multiple steps in TPM equal to 12^55^. The Wilcoxon test was used to depict significant heterozygosity deficiency and only the resulting P values were presented in Table 1. Isolation by distance (IBD) among populations was tested using a Mantel test implemented in IBD v. 1.5^56^ to test for an association between linearised pairwise population genetic differences (*F*ST/(1 − *F*ST)) and coastal distance.

**Table 1:**
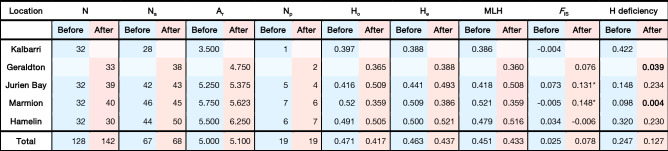
Metrics of genetic diversity before (blue) and after (red) the marine heatwave.

*N* number of samples, *N*_*a*_ the total number of alleles, *A*_*r*_ the allelic richness, *N*_*p*_ the number of private alleles, *H*_*o*_ observed heterozygosity, *H*_*e*_ expected heterozygosity, *MLH* mean number of heterozygous loci per individual. *F*_IS_ = inbreeding coefficient and H deficiency is the P value for test of heterozygote deficiency indicative of a population expansion. No data was available at Kalbarri after the heatwave because populations of *Ecklonia* were extirpated. Instead, the new range edge (Geraldton) was sampled after the heatwave.

## Results

Contrary to our prediction, there was no wholesale loss of genetic diversity within the footprint of the marine heatwave, with all diversity metrics remaining relatively constant (Table 1). The exception was genetic diversity (measured as expected heterozygosity and MLH) at Marmion which suffered a 24 and 31% decline respectively (Table 1). Diversity at the new range edge (Geraldton; only sampled after) was one of the lowest and was similar to the old range edge (Kalbarri) where kelp was completely extirpated. Jurien Bay and Hamelin both slightly increased genetic diversity by 4–12% (He) and 21–7% (MLH) respectively due to a gain of alleles overall. After the heatwave, Hamelin gained several new alleles that were previously only found in low latitude sites (e.g. EC10 allele 236, EC12 allele 169 and Eradic 10 allele 451 and 454; Supplementary Table S1). Jurien Bay gained 7 new alleles after the heatwave which were previously characteristic of both low and higher latitude sites. The presence of private alleles both before and after the heatwave may be used as an indicator of population stability. At Hamelin, 43% of private alleles remained after the heatwave relative to only 17–25% at Marmion and Jurien Bay (Table 1; Supplementary Table S1). The inbreeding coefficient (*F*_IS_) became significantly positive at Marmion and Jurien Bay after the heatwave indicating that these sites became inbred (Table 1). Moreover, at Marmion there was a significant deficiency of heterozygotes after the heatwave indicative of recent population expansion following a ~ 25% cover loss^6^.

**Figure 3:**
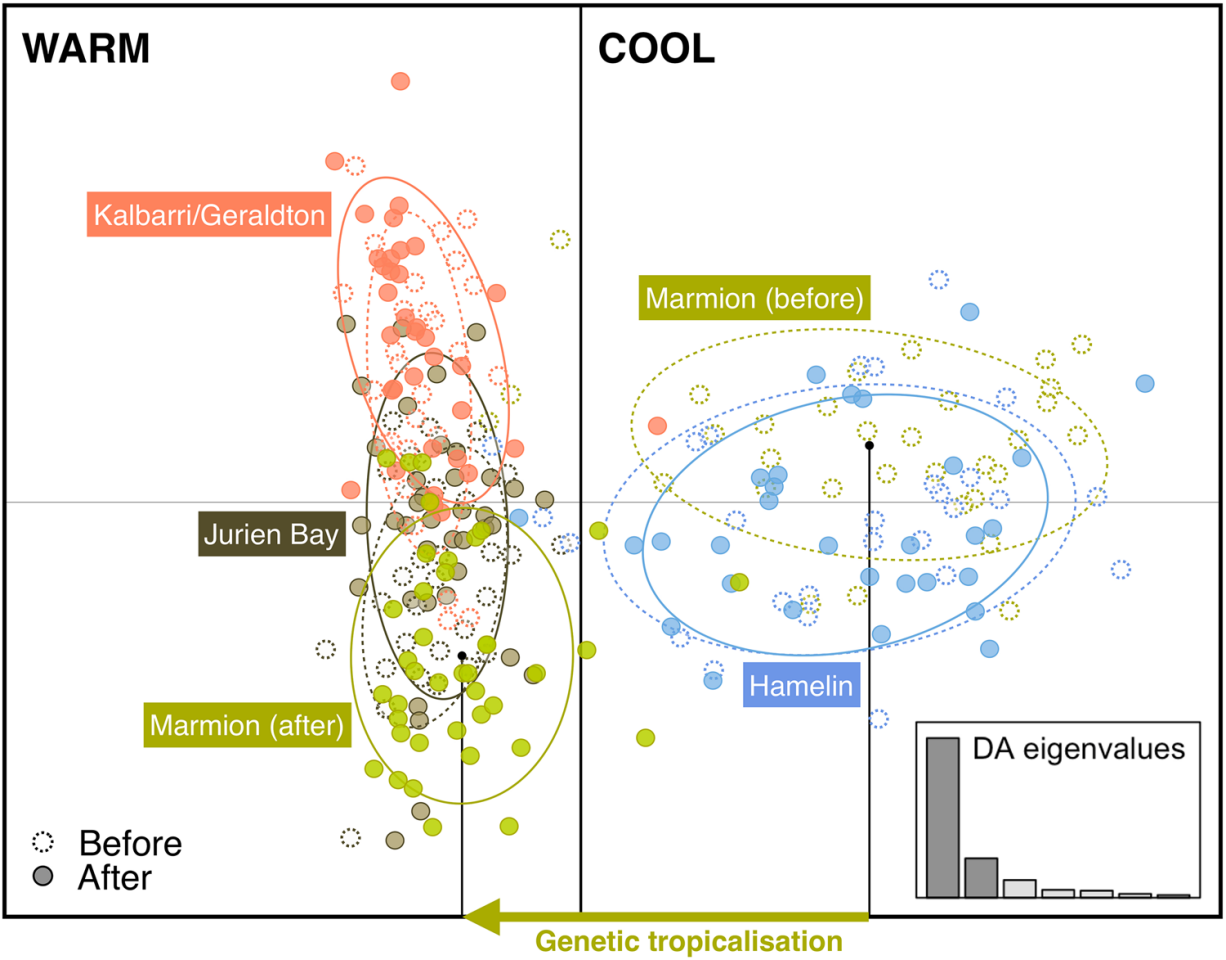
Discriminant analysis of principal components (DAPC) before (dashed symbols) and after (solid symbols) the heatwave. Each dot represents an individual while populations are represented by a coloured circle (the circumference of the ellipsis is arbitrary). The amount of genetic variation explained by the first two linear discriminants (DA) are 67.01 and 16.54%.

Populations of kelp contained 2 genetic clusters both before and after the heatwave (Fig. 2; K = 2 inferred from delta K). However, the spatial composition of those clusters changed after the heatwave. Marmion shifted from being within a “cool” genetic cluster of genotypes characteristic of high latitude sites to an inbred “warm” allele cluster characteristic of genotypes from low latitude sites (Table 1, Figs. 2, 3) after the heatwave. That is, prior to the heatwave, Marmion grouped with the other high latitude (cool) site (Hamelin) but after the heatwave it became part of the low latitude (warm) cluster containing Jurien Bay and Geraldton (Figs. 2, 3). As such, there was significant genetic differentiation at Marmion from before to after the heatwave (*F*_ST_ = 0.112) and this temporal difference was ~ 8 times greater than the same temporal comparison between any other pair of sites (Table 2).

**Table 2:**
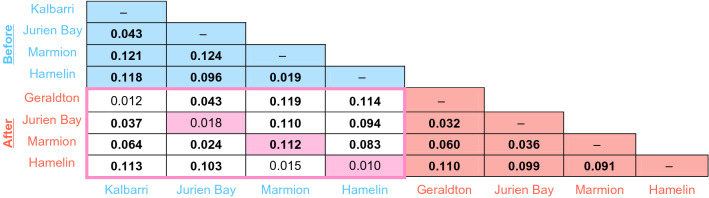
Pairwise *F*_ST_ estimates between pairs of sites before (blue) and after (red) the heatwave as well as comparisons between each site (pink) and different sites (no shading) before to after the heatwave event.

Values in bold are significant after the Bonferroni correction (P < 0.001). Values in bold are significant after the Bonferroni correction (P < 0.001).

The DAPC plot mirrors this pattern showing that Marmion shifted to being more closely related to Jurien Bay and Kalbarri (before) and Geraldton after the heatwave. Moreover, Jurien Bay appears to have become slightly more similar to Kalbarri/Geraldton in the DAPC plot after the heatwave (overlapping inertia ellipses; Fig. 3) concurrent with a drop in the pairwise *F*_ST_ estimate between these sites (Table 2) indicating that the kelp population at Jurien Bay became more genetically similar to its low latitude (warm) neighbours. There was also a slight temporal shift in genetic differentiation at Jurien Bay from before to after the heatwave (significant only at P < 0.01, Table 2). Kalbarri/Geraldton and Hamelin did not show any significant temporal change in genetic differentiation from before to after the heatwave (Table 2). The pattern of isolation by distance (IBD) became highly significant after the heatwave (Mantel tests Z = 691.70, r = 0.92, P < 0.001) but was not significant before (Mantel tests Z = 500.99, r = 0.40, P = 0.17).

**Figure 4:**
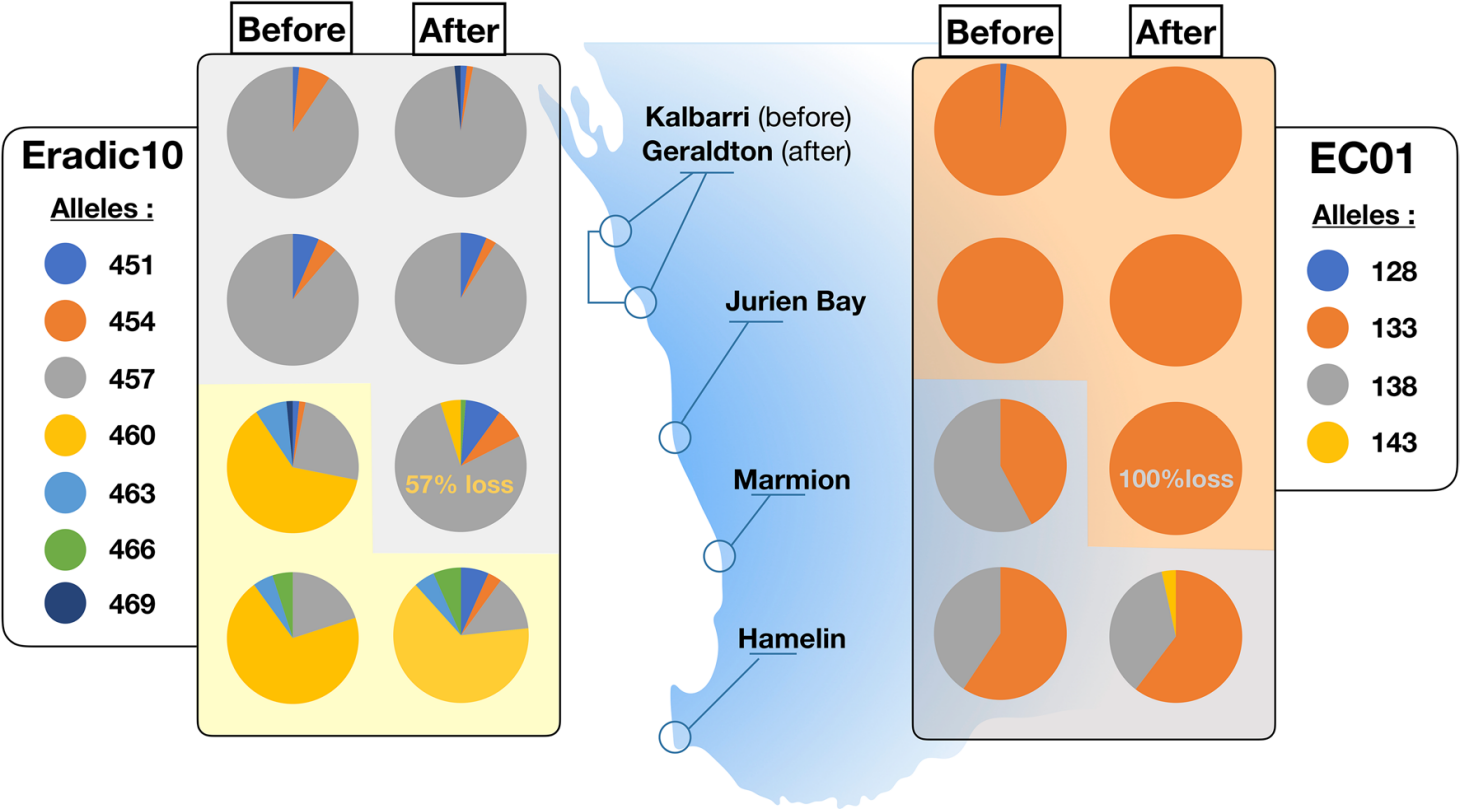
Examples of change in allelic frequencies of two microsatellite loci contributing to genetic tropicalisation at Marmion [Eradic10 (*n* = 26–40) and EC01 (*n* = 30–40)]. Background shading represents the northern and southern clusters identified from STRUCTURE (v 2.3.4; https://web.stanford.edu/group/pritchardlab/structure) (K = 2; Fig. 3) and are coloured according to the major allele frequency driving change from before to after. The percent loss of the major “cool” alleles (460 and 138) at Marmion is also shown.

This striking shift at Marmion from the cool to warm genotype cluster was driven by clear shifts in allele frequencies at many loci (Fig. 3, Supplementary Fig. S1). Notably, Eradic10 had the greatest contribution to separation of warm and cool clusters on DAPC axes (Fig. 3, Supplementary Fig. S1). For this locus, allele 457, which was largely characteristic of the low latitude (warm) cluster (Kalbarri/Jurien Bay), became the dominant allele at Marmion after the heatwave, concurrent with a decline in the frequency of allele 460, which was overwhelmingly dominant in the higher latitude (cool) cluster before (Marmion and Hamelin) and after (Hamelin) the heatwave (Fig. 4). Similarly, alleles 133 and 138 of EC01 showed significant change at Marmion after the heatwave (Fig. 3, Supplementary Table S3) and allele 184 of EC12 also contributed to the shift at Marmion (Supplementary Table S3). Locus by locus AMOVA confirmed that Eradic10 and EC01 loci drove 42–50% (Eradic10) and 31–49% (EC01) of genetic variation between the 2 STRUCTURE clusters before and after the heatwave (Supplementary Material, Supplementary Table S2).

## Discussion

Marine heatwaves are increasing globally with devastating ecological consequences, but the impacts on underlying genetics of species and populations are poorly understood. Using very rare “before” genetic samples we empirically demonstrate that an extreme marine heatwave was associated with significant change in kelp forest genetic diversity and structure. Contrary to our prediction, there was no wholesale loss of genetic diversity within the footprint of the marine heatwave despite great loss of kelp in some sites. Along the entire coast of Western Australia, however, the heatwave drove kelp populations to become more genetically distinct with a significant increase in isolation by distance. Moreover, the heatwave led to replacement of cool-water alleles by warm-water alleles across 200 km of coastline, representing a clear and unprecedented “genetic tropicalisation”.

### Genetic tropicalisation of kelp forests

Tropicalisation is traditionally defined as an increase in relative abundance of warm-water species^24^ in cool-water ecological communities and is a well-documented response to warming in the ecological literature^57–59^, including in this study area following the marine heatwave^6^. However, to our knowledge this is the first time that a similar phenomenon has been demonstrated to extend to the molecular level. After the heatwave, Marmion shifted from a “cool” genetic cluster characteristic of high latitude sites to an inbred “warm” genetic cluster characteristic of low latitude sites. This striking change was driven by clear shifts in allele frequencies at a number of loci, whereby alleles largely characteristic of the low latitude (warm) cluster prior to the heatwave, became the dominant alleles at Marmion after the heatwave, concurrent with a decline in alleles which were overwhelmingly dominant in the higher latitude (cool) cluster. Thus, we define these changes as “genetic tropicalisation”. This phenomenon is a specific case of a hybrid zone, whereby populations at the interface of species or genetic clusters periodically mix to various degrees^60–62^ which can also be climate mediated^62^.

Genetic tropicalisation at Marmion was likely facilitated by mortality of kelp that occurred as a direct result of the heatwave^6^. Kelp recruitment is often low within dense canopies and turnover largely reliant on the change in physical conditions created as kelp density thins or gaps are created following mortality or loss of adult plants^63,64^. Indeed, Marmion initially suffered ~ 25% decline in kelp cover due to the direct physiological effects of the heatwave^6^ which likely exceeded or approached the thermal tolerance of kelp and co-occurring seaweed species^6,65,66^. This opened up space for rapid recruitment by either (1) locally surviving ‘warm’ genotypes^20^ or (2) propagules transported from lower latitudes assisted by the increased poleward flow of prevailing currents^35,67^ at the time of the heatwave^31^. These scenarios of recolonization are not mutually exclusive and likely occurred together, although our microsatellite data can only provide definitive evidence for the latter. If genetic tropicalisation was a result of proliferation of surviving warm genotypes at Marmion it would imply there was selection against ‘cool’ genotypes. While this may indeed have occurred, selection cannot be inferred from neutral microsatellite data. Nevertheless, Marmion suffered the greatest loss of genetic diversity of any site (24–31% of expected and multi-locus heterozygosity), suggesting that there could have been some selection. Moreover, Marmion became inbred after the heatwave suggesting that breeding was among a smaller pool of surviving genotypes rather than a large influx of new ones. Regardless of the mechanism driving the change, the timing of the heatwave during the peak reproductive season for *Ecklonia*^68^, meant that the genetic tropicalisation signal was likely rapidly entrenched. The rapid recovery of kelp canopy cover at Marmion would have prevented any further major genetic turnover because the ecological driver of such turnover (gaps in the canopy that facilitate recruitment of local or foreign genotypes) was removed. Indeed, this genetic tropicalisation signal was evident even 7 years after the heatwave.

For other sites for which we had before and after data (Hamelin and Jurien Bay) there was only a small genetic effect of the heatwave. Genetic diversity at Hamelin slightly increased, gaining a number of low frequency alleles characteristic of low latitude (warm) sites. Despite experiencing similarly large thermal anomalies, we contend that Hamelin only experienced mild genetic change because there was largely no mechanism for this process to occur. Hamelin did not experience significant loss of kelp during the heatwave presumably because its thermal threshold was not crossed^24^ nor has it shown any evidence of ecological tropicalisation^6,24^, implying that no space was opened up for significant genetic turnover to occur. Indeed, the relatively greater stability of private alleles at Hamelin and presence of the lowest pairwise F_ST_ estimates between times of sampling indicate little genetic turnover at this site.

Interestingly, there was only a small genetic effect of the heatwave at Jurien Bay, despite great loss of kelp cover (50–60%) and strong ecological tropicalisation^6,24^. While this loss provides a mechanism for genetic turnover, a large shift in neutral alleles could not take place as Jurien Bay was already part of the low latitude cluster and dispersal along this coast is predominately poleward^35,67^ with few remaining populations further north. Thus, recolonization following loss of kelp would not leave a genetic signature because recruits would most likely be sourced from within the same genetic cluster. Nevertheless, *F*_IS_ became significant at Jurien Bay after the heatwave indicating inbreeding and Jurien Bay became slightly more similar to Kalbarri/Geraldton on the DAPC plot after the heatwave concurrent with a drop in the pairwise *F*_ST_ estimate between these sites indicating that the kelp population at Jurien Bay became slightly more genetically similar to its low latitude (warm) trailing edge neighbours, possibly a mild tropicalisation signal.

Although the correlative nature of this study and lack of more temporal replication means that we cannot definitively demonstrate that the heatwave caused the genetic changes seen here, genetic change seen in other seaweed species and markers following the heatwave is strong evidence to suggest that the heatwave did mediate genetic tropicalisation^20^. Two co-occurring forest forming species *Scytothalia dorycarpa* and *Sargassum fallax*^29^, both showed strong declines in haplotype diversity following the heatwave^20^ with *Scytothalia* being completely extirpated from Jurien Bay^25^. Moreover, there were some signs of possible directional selection for *Sargassum* with specific genotypes dominating after the heatwave^20^. Nevertheless, genetic change from before to after the heatwave could also be simply due to one of many temporally stochastic processes that influence genetic turnover in populations. For example, temporal variation in oceanography^69^, reproductive success^70^, ecological processes (e.g. herbivory) or post-recruitment survival of different genotypes may result in significant temporal genetic change. Unfortunately there exist very few temporally replicated genetic datasets, particularly for seaweeds, to tease apart the role of such stochastic processes in driving genetic change^71^. The few existing temporally replicated seaweed studies show contrasting results. For example, *Fucus serratus* populations sampled 10 years apart across Europe, showed temporal genetic stability^48^ except in warm range edge populations that were thermally stressed. These populations experienced a 90% decline in abundance and greater homozygosity (decline in MLH), possibly as a result of selection favouring thermal tolerance^48^. Conversely, an extreme event (earthquake) in Chile resulted in a decline in *Agarophyton chilense* genetic diversity (loss of rare alleles) immediately following the event, but recovery occurred after 2 years^72^. Given the weight of evidence we present here and across multiple species and genetic markers^20^, we find it likely the heatwave was responsible for the observed genetic changes. However, proper understanding of the genetic impacts of climate change and extreme events for marine organisms will require temporally replicated studies and efforts to collect baseline data from which to measure change.

Kelps possess a microscopic gametophyte stage which may be able to tolerate different environmental conditions than sporophytes and potentially act as a short term ‘bank of microscopic forms’ for recolonisation following stressors^73^. Genetic studies have shown that these banks can also be of mixed origin and age which maximises genetic diversity of recolonising populations^74^. Given that gametophytes of *E. radiata* appear to have broad thermal tolerances with lethal upper limits that are generally in excess of temperatures experienced in the field^30^, it is possible that recovery following heatwave-induced mortality of macroscopic sporophytes was partly from such a bank of microscopic forms. Recovery from a genetically diverse, mixed age or origin bank could explain the general maintenance of genetic diversity across most sites, even where there was large loss of canopy cover (Jurien). Moreover, recovery from an existing bank at Marmion may have dampened the impact of increased gene flow from lower latitudes. However, the difficulties in finding and sampling gametophytes in natural settings mean that large knowledge gaps remain regarding their role in population recovery following extreme events and kelp forest population genetics more generally.

### The future of kelp forests

It is interesting to speculate whether populations at Marmion are now more thermally tolerant than before the heatwave. Properly identifying adaptation in complex natural systems is a challenging task^75,76^ and is outside the scope of this study. Nonetheless, while our study used neutral microsatellite markers and can only definitively infer change in gene flow, it is plausible that, because lower latitude populations had a greater thermal tolerance than higher latitude populations prior to the heatwave^77^, this greater tolerance would now be seen in Marmion due to an increase in low latitude “warm” genotypes. Given that reduction in temperature sensitivity of metabolic processes from cool- to warm-adapted populations has previously been demonstrated for several seaweed species along this coastline^77^ (a mechanisms to help constrain temperature-driven effects on net carbon balance), we find this scenario likely. Unravelling whether the changes seen here in neutral microsatellite markers are reflected in those under selection (adaptive markers), along with manipulative experiments to assess thermal tolerance of different genotypes will be key for predicting future vulnerability of these kelp forests to ongoing climate change.

It has been 9 years since the marine heatwave and while the lowest latitude kelp forests in Kalbarri have not yet recovered^78^, genetic signs of population expansion at Geraldton and Marmion (Table 1) suggest that recovery and recolonization at these sites is ongoing and a long-term process. While encouraging, the frequency of marine heatwaves is projected to increase^12^ and warm low latitude populations remain somewhat vulnerable due to inbreeding coupled with genetic erosion^79^, factors which may undermine long term adaptive capacity.

Global kelp loss has prompted calls for more interventionist management approaches to actively ensure long-term persistence against climate change^80,81^. In the case of Australian kelp forests, the use of interventionist measures may be one of the few routes left to address the alarming predicted range contraction of *Ecklonia* and extinction of other seaweeds^80,82,83^. These strategies include assisted adaptation and evolution, which are founded on the concept that incorporating stress tolerant genes or genotypes into extant populations can boost their resilience to future change^83^. This highlights a pressing need to identify where and when one may find such tolerant individuals and populations to apply such approaches. We suggest that extreme events may provide a natural laboratory for identifying tolerant populations or genotypes that could be targeted and utilised in assisted adaptation approaches. Recognising these potentially positive benefits of extreme events may present new opportunities to “future-proof” ecosystems against climate change.

## Supplementary information

Supplementary file1

## References

[1] Sath IPCC (2014). Climate Change 2014: Synthesis Report. Contribution of Working Groups I, II and III to the Fifth Assessment Report of the Intergovernmental Panel on Climate Change.

[2] Coumou D, Rahmstorf S (2012). A decade of weather extremes. Nat. Clim. Change.

[3] Grant PR (2017). Evolution caused by extreme events. Philos. Trans. R. Soc. B..

[4] Jangjoo M, Matter SF, Roland J, Keyghobadi N (2016). Connectivity rescues genetic diversity after a demographic bottleneck in a butterfly population network. Proc. Natl. Acad. Sci. USA.

[5] Gaines SD, Denny MW (1993). The largest, smallest, highest, lowest, longest, and shortest: Extremes in ecology. Ecology.

[6] Wernberg T (2016). Climate-driven regime shift of a temperate marine ecosystem. Science.

[7] Vincenzi S, Mangel M, Jesensek D, Garza JC, Crivelli AJ (2017). Genetic and life-history consequences of extreme climate events. Proc. R. Soc. B Biol. Sci..

[8] Poff NL (2018). Extreme streams: Species persistence and genomic change in montane insect populations across a flooding gradient. Ecol. Lett..

[9] Campbell-Staton SC (2017). Winter storms drive rapid phenotypic, regulatory, and genomic shifts in the green anole lizard. Science.

[10] Wernberg T (2018). Genetic diversity and kelp forest vulnerability to climatic stress. Sci. Rep. Uk.

[11] Hobday AJ (2016). A hierarchical approach to defining marine heatwaves. Prog. Oceanogr..

[12] Oliver ECJ (2018). Longer and more frequent marine heatwaves over the past century. Nat. Commun..

[13] Smale DA (2019). Marine heatwaves threaten global biodiversity and the provision of ecosystem services. Nat. Clim. Change.

[14] Straub SC (2019). Resistance, extinction, and everything in between—the diverse responses of seaweeds to marine heatwaves. Front. Mar. Sci..

[15] Arafeh-Dalmau N (2019). Extreme marine heatwaves alter kelp forest community near its equatorward distribution limit. Front. Mar. Sci..

[16] Thomsen MS (2019). Local extinction of bull kelp (*Durvillaea* spp.) due to a marine heatwave. Front. Mar. Sci..

[17] Rogers-Bennett L, Catton CA (2019). Marine heat wave and multiple stressors tip bull kelp forest to sea urchin barrens. Sci. Rep. Uk.

[18] Bell TM, Strand AE, Sotka EE (2014). The adaptive cline at LDH (Lactate Dehydrogenase) in Killifish *Fundulus heteroclitus* remains stationary after 40 years of warming Estuaries. J. Hered..

[19] Hilbish TJ (2012). Change and stasis in marine hybrid zones in response to climate warming. J. Biogeogr..

[20] Gurgel CFD, Camacho O, Minne AJP, Wernberg T, Coleman MA (2020). Marine heatwave drives cryptic loss of genetic diversity in underwater forests. Curr. Biol..

[21] Reusch TBH, Ehlers A, Hämmerli A, Worm B (2005). Ecosystem recovery after climatic extremes enhanced by genotypic diversity. Proc. Natl. Acad. Sci. USA.

[22] Hobday AJ (2018). Categorizing and naming marine heatwaves. Oceanography.

[23] Pearce, A. *et al.**The “Marine Heat Wave” off Western Australia During the Summer of 2010/11*. (Government of Western Australia, Department of Fisheries., Western Australia, 2011).

[24] Wernberg T (2013). An extreme climatic event alters marine ecosystem structure in a global biodiversity hotspot. Nat. Clim. Change.

[25] Smale DA, Wernberg T (2013). Extreme climatic event drives range contraction of a habitat-forming species. Proc. R. Soc. B Biol. Sci..

[26] Moore JAY (2012). Unprecedented mass bleaching and loss of coral across 12° of latitude in Western Australia in 2010–11. PLoS One.

[27] Caputi N (2016). Management adaptation of invertebrate fisheries to an extreme marine heat wave event at a global warming hot spot. Ecol. Evol..

[28] Bennett S (2016). The 'Great Southern Reef': Social, ecological and economic value of Australia's neglected kelp forests. Mar. Freshw. Res..

[29] Coleman MA, Wernberg T (2017). Forgotten underwater forests: The key role of fucoids on Australian temperate reefs. Ecol. Evol..

[30] Wernberg T (2019). Biology and ecology of the globally significant kelp *Ecklonia radiata*. Oceanogr. Mar. Biol. Annu. Rev..

[31] Feng M, McPhaden MJ, Xie S-P, Hafner JL (2013). Niña forces unprecedented Leeuwin current warming in 2011. Sci. Rep. Uk.

[32] Coleman MA, Vytopil E, Goodsell PJ, Gillanders BM, Connell SD (2007). Diversity and depth-related patterns of mobile invertebrates associated with kelp forests. Mar. Freshw. Res..

[33] Coleman MA, Kennelly SJ (2019). Microscopic assemblages in kelp forests and urchin barrens. Aquat. Bot..

[34] Coleman MA, Wernberg T (2018). Genetic and morphological diversity in sympatric kelps with contrasting reproductive strategies. Aquat. Biol..

[35] Coleman MA (2011). Variation in the strength of continental boundary currents determines continent-wide connectivity in kelp. J. Ecol..

[36] Little AG, Fisher DN, Schoener TW, Pruitt JN (2019). Population differences in aggression are shaped by tropical cyclone-induced selection. Nat. Ecol. Evol..

[37] Schiebelhut LM, Puritz JB, Dawson MN (2018). Decimation by sea star wasting disease and rapid genetic change in a keystone species, *Pisaster ochraceus*. Proc. Natl. Acad. Sci..

[38] Coleman MA, Gillanders BM, Connell SD (2009). Dispersal and gene flow in the habitat-forming kelp, *Ecklonia radiata*: Relative degrees of isolation across an east–west coastline. Mar. Freshw. Res..

[39] Coleman MA (2011). Connectivity within and among a network of temperate marine reserves. PLoS One.

[40] Itou T (2012). Development of 12 polymorphic microsatellite DNA markers for the kelp Ecklonia cava (Phaeophyceae, Laminariales). Conserv. Genet. Resour..

[41] Akita S (2018). Development of 11 Ecklonia radicosa (Phaeophyceae, Laminariales) SSRs markers using next-generation sequencing and intra-genus amplification analysis. J. Appl. Phycol..

[42] Van Oosterhout C, Hutchinson WF, Wills DPM, Shipley P (2004). micro-checker: Software for identifying and correcting genotyping errors in microsatellite data. Mol. Ecol. Notes.

[43] Excoffier L, Lischer HEL (2010). Arlequin suite ver 3.5: A new series of programs to perform population genetics analyses under Linux and Windows. Mol. Ecol. Resour..

[44] Agapow P-M, Burt A (2001). Indices of multilocus linkage disequilibrium. Mol. Ecol. Notes.

[45] Guo SW, Thompson EA (1992). Performing the exact test of Hardy–Weinberg proportion for multiple alleles. Biometrics.

[46] Peakall R, Smouse PE (2012). GenAlEx 6.5: Genetic analysis in Excel. Population genetic software for teaching and research—an update. Bioinformatics.

[47] Belkhir, K., Borsa, P., Chikhi, L., Raufaste, N. & Bonhomme, F. *GENETIX 402, logiciel sous Windows TM pour la génétique des populations* (2000).

[48] Jueterbock A, Coyer JA, Olsen JL, Hoarau G (2018). Decadal stability in genetic variation and structure in the intertidal seaweed *Fucus serratus* (Heterokontophyta: Fucaceae). BMC Evol. Biol..

[49] Goudet J (1995). FSTAT (Version 1.2): A computer program to calculate F-statistics. J. Hered..

[50] Evanno G, Regnaut S, Goudet J (2005). Detecting the number of clusters of individuals using the software STRUCTURE: A simulation study. Mol. Ecol..

[51] Earl DA, von Holdt BM (2012). STRUCTURE HARVESTER: A website and program for visualizing STRUCTURE output and implementing the Evanno method. Conserv. Genet. Resour..

[52] Francis RM (2017). Pophelper: An R package and web app to analyse and visualize population structure. Mol. Ecol. Resour..

[53] Jombart T (2008). Adegenet: A R package for the multivariate analysis of genetic markers. Bioinformatics.

[54] Luikart G, Cornuet J-M (1998). Empirical evaluation of a test for identifying recently bottlenecked populations from allele frequency data. Conserv. Biol..

[55] Cornuet JM, Luikart G (1996). Description and power analysis of two tests for detecting recent population bottlenecks from allele frequency data. Genetics.

[56] Bohonak AJ (2002). IBD (isolation by distance): A program for analyses of isolation by distance. J. Hered..

[57] Vergés A (2016). Long-term empirical evidence of ocean warming leading to tropicalization of fish communities, increased herbivory, and loss of kelp. Proc. Natl. Acad. Sci..

[58] Vergés A (2014). The tropicalization of temperate marine ecosystems: Climate-mediated changes in herbivory and community phase shifts. Proc. R. Soc. B-Biol. Sci..

[59] Vergés A (2019). Tropicalisation of temperate reefs: Implications for ecosystem functions and management actions. Funct. Ecol..

[60] Wielstra B (2019). Historical hybrid zone movement: More pervasive than appreciated. J. Biogeogr..

[61] Taylor SA (2014). Climate-mediated movement of an avian hybrid zone. Curr. Biol..

[62] Taylor SA, Larson EL, Harrison RG (2015). Hybrid zones: Windows on climate change. Trends Ecol. Evol..

[63] Wernberg T, Connell SD (2008). Physical disturbance and subtidal habitat structure on open rocky coasts: Effects of wave exposure, extent and intensity. J. Sea Res..

[64] Wernberg T (2010). Decreasing resilience of kelp beds along a latitudinal temperature gradient: Potential implications for a warmer future. Ecol. Lett..

[65] Bennett S, Wernberg T, Joy BA, De Bettignies T, Campbell AH (2015). Central and rear-edge populations can be equally vulnerable to warming. Nat. Commun..

[66] Wernberg T, de Bettignies T, Bijo AJ, Finnegan P (2016). Physiological responses of habitat-forming seaweeds to increasing temperatures. Limnol. Oceanogr..

[67] Coleman MA, Feng M, Roughan M, Cetina-Heredia P, Connell SD (2013). Temperate shelf water dispersal by Australian boundary currents: Implications for population connectivity. Limnol. Oceanogr. Fluids Environ..

[68] Mohring MB, Wernberg T, Kendrick GA, Rule MJ (2013). Reproductive synchrony in a habitat-forming kelp and its relationship with environmental conditions. Mar. Biol..

[69] Barshis DJ (2011). Coastal upwelling is linked to temporal genetic variability in the acorn barnacle *Balanus glandula*. Mar. Ecol. Prog. Ser..

[70] Planes S, Lenfant P (2002). Temporal change in the genetic structure between and within cohorts of a marine fish, *Diplodus sargus*, induced by a large variance in individual reproductive success. Mol. Ecol..

[71] Toonen, R. J. & Grosberg, R. K. *Phylogeography and Population Genetics in Crustacea* (eds S. Koenemann, C. Held, & C. Schubart) 75–107 (CRC Press, Boca Raton, 2011).

[72] Becheler R (2020). After a catastrophe, a little bit of sex is better than nothing: Genetic consequences of a major earthquake on asexual and sexual populations. Evol. Appl..

[73] Hoffmann AJ, Santelices B (1991). Banks of algal microscopic forms: Hypotheses on their functioning and comparisons with seed banks. Mar. Ecol. Prog. Ser..

[74] Carney LT, Bohonak AJ, Edwards MS, Alberto F (2013). Genetic and experimental evidence for a mixed-age, mixed-origin bank of kelp microscopic stages in southern California. Ecology.

[75] Hoban S (2016). Finding the genomic basis of local adaptation: Pitfalls, practical solutions, and future directions. Am. Nat..

[76] Pardo-Diaz C, Salazar C, Jiggins CD (2015). Towards the identification of the loci of adaptive evolution. Methods Ecol. Evol..

[77] Staehr PA, Wernberg T (2009). Physiological responses of Ecklonia Radiata (Laminariales) to a latitudinal gradient in ocean temperature. J. Phycol..

[78] Wernberg, T. in *Ecosystem Collapse and Climate Change.* (eds Canadell JG & Jackson RB) (Springer-Nature, 2020).

[79] Nicastro KR (2013). Shift happens: Trailing edge contraction associated with recent warming trends threatens a distinct genetic lineage in the marine macroalga *Fucus vesiculosus*. BMC Biol..

[80] Coleman MA, Goold H (2019). Harnessing synthetic biology for kelp forest conservation. J. Phycol..

[81] Wood G (2019). Restoring subtidal marine macrophytes in the Anthropocene: Trajectories and future-proofing. Mar. Freshw. Res..

[82] Martínez B (2018). Predictions of responses to ocean warming for habitat-forming seaweeds. Divers. Distrib..

[83] Coleman M (2020). Restore or redefine: Future trajectories for restoration. Front. Mar. Sci..

