# Supplementary material for: Genetic tropicalisation following a marine heatwave
Source: Sci Rep. 2020 Jul 29;10:12726. doi: 10.1038/s41598-020-69665-w (PMC7391769; doi:10.1038/s41598-020-69665-w)

**Supplementary Material**

**Genetic tropicalisation following a marine heatwave**

Melinda A. Coleman, Antoine J. P. Minne, Sofie Vranken and Thomas Wernberg

**Table S1.** Characteristics of the eight selected polymorphic genomic microsatellite loci used for *Ecklonia radiata*. Markers were tested and chosen based on those developed for *E. cava* (Itou et al., 2012) and *E. radicosa* (Akita et al., 2018).


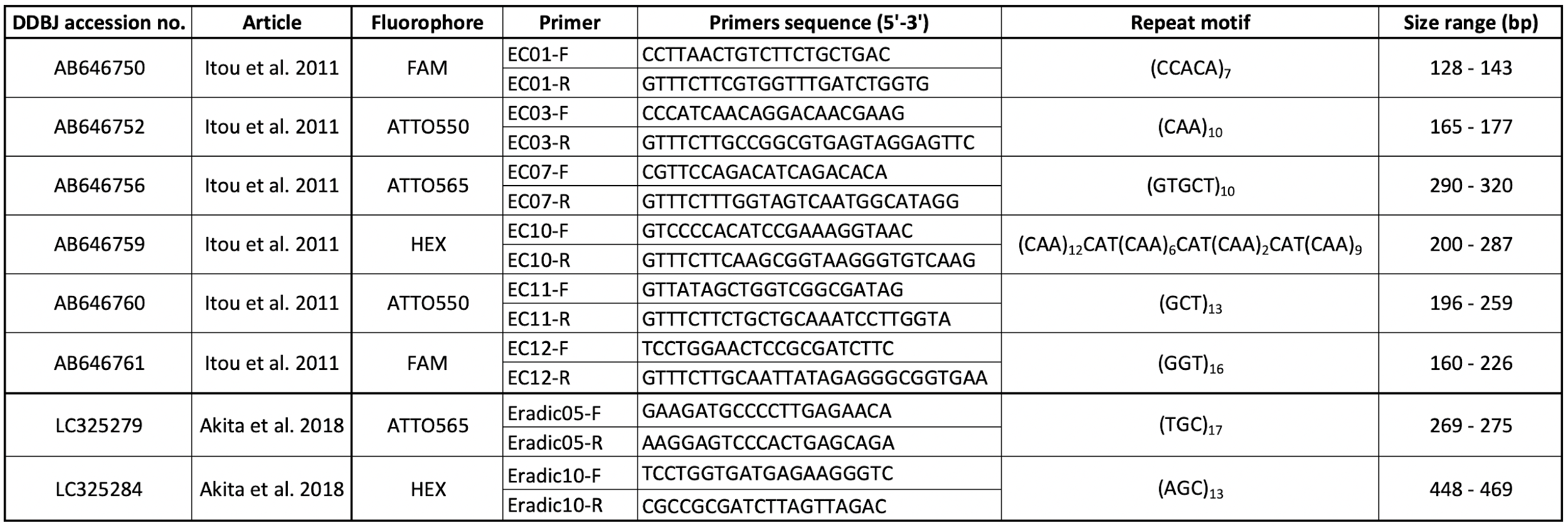


**Table S2.** Global Analysis of Molecular Variance (AMOVA) of spatial genetic variation in *Ecklonia radiata* for eight microsatellites. *** = P < 0.0001. The percentage of variation (from the locus by locus AMOVA) explained by the 2 main loci responsible for the genetic tropicalisation and shift in genetic clusters is also shown.

|  | | d.f. | | SS | | Variance component | | % variation | |  | Eradic10  % variation | | Eco1  % variation | |
| --- | --- | --- | --- | --- | --- | --- | --- | --- | --- | --- | --- | --- | --- | --- |
| **BEFORE** | |  | |  | |  | |  | |  |  | |  | |
| Between clusters | | 2 | | 51.80 | | 0.36 | | 15.55 | | *** | 56.10 | | 46.56 | |
| Among population within clusters | | 2 | | 13.30 | | 0.08 | | 3.42 | | *** |  | |  | |
| Among individuals within populations | | 138 | | 224.83 | | 0.04 | | 1.89 | |  |  | |  | |
| Within individuals | | 142 | | 221.50 | | 1.83 | | 79.14 | | *** |  | |  | |
|  | |  | |  | |  | |  | |  |  | |  | |
| **AFTER** | |  | |  | |  | |  | |  |  | |  | |
| Between clusters | | 2 | | 34.17 | | 0.27 | | 12.15 | | *** | 60.73 | | 58.07 | |
| Among population within clusters | | 2 | | 21.67 | | 0.12 | | 5.54 | | *** |  | |  | |
| Among individuals within populations | | 138 | | 253.83 | | 0.08 | | 3.48 | |  |  | |  | |
| Within individuals | | 142 | | 240.5 | | 1.73 | | 78.83 | | *** |  | |  | |
|  |  | |  | |  | |  | |  |  | |  | |  |

**Table S3.** Allele frequencies before and after the heatwave for all loci with *F*_IS_ values (before/after) into brackets under each locus name. Site abbreviations are Kalbarri (KAL), Geraldton (GER), Jurien Bay (JUR), Marmion (MAR) and Hamelin (HAM).

|  |  | **BEFORE** | | | | **AFTER** | | | |
| --- | --- | --- | --- | --- | --- | --- | --- | --- | --- |
| **Locus (*F*_IS_)** | **Allele** | **KAL** | **JUR** | **MAR** | **HAM** | **GER** | **JUR** | **MAR** | **HAM** |
| **EC01** | **128** | 0.016 | 0.000 | 0.000 | 0.000 | **0.000** | 0.000 | 0.000 | 0.000 |
| (-0.046/0.044) | **133** | 0.984 | 1.000 | 0.422 | 0.594 | 1.000 | 1.000 | 1.000 | 0.603 |
|  | **138** | 0.000 | 0.000 | 0.578 | 0.406 | 0.000 | 0.000 | **0.000** | 0.362 |
|  | **143** | 0.000 | 0.000 | 0.000 | 0.000 | 0.000 | 0.000 | 0.000 | 0.034 |
| **ECO3** | **165** | 0.000 | 0.000 | 0.016 | 0.000 | 0.000 | 0.000 | **0.000** | 0.000 |
| (-0.011/0.145) | **168** | 0.094 | 0.203 | 0.078 | 0.194 | 0.063 | 0.244 | 0.138 | 0.183 |
|  | **171** | 0.906 | 0.688 | 0.844 | 0.774 | 0.938 | 0.654 | 0.825 | 0.750 |
|  | **174** | 0.000 | 0.109 | 0.063 | 0.032 | 0.000 | 0.090 | 0.038 | 0.067 |
|  | **177** | 0.000 | 0.000 | 0.000 | 0.000 | 0.000 | **0.013** | 0.000 | 0.000 |
| **EC07** | **290** | 0.000 | 0.000 | 0.016 | 0.000 | 0.000 | 0.000 | **0.000** | **0.034** |
| (0.007/-0.111) | **305** | 0.179 | 0.323 | 0.048 | 0.130 | 0.094 | 0.192 | 0.038 | 0.069 |
|  | **310** | 0.821 | 0.629 | 0.919 | 0.815 | 0.750 | 0.615 | 0.863 | 0.879 |
|  | **315** | 0.000 | 0.048 | 0.000 | 0.056 | **0.156** | 0.192 | **0.088** | 0.017 |
|  | **320** | 0.000 | 0.000 | 0.016 | 0.000 | 0.000 | 0.000 | 0.013 | 0.000 |
| **EC10** | **200** | 0.032 | 0.033 | 0.000 | 0.000 | 0.065 | 0.026 | 0.000 | 0.000 |
| (0.123/0.100) | **203** | 0.113 | 0.233 | 0.031 | 0.050 | 0.016 | 0.179 | 0.275 | 0.052 |
|  | **206** | 0.000 | 0.000 | 0.000 | 0.000 | **0.016** | 0.000 | **0.013** | 0.000 |
|  | **209** | 0.000 | 0.000 | 0.000 | 0.000 | **0.016** | 0.000 | 0.000 | 0.000 |
|  | **218** | 0.000 | 0.000 | 0.000 | 0.000 | 0.000 | 0.000 | 0.025 | 0.000 |
|  | **224** | 0.000 | 0.017 | 0.000 | 0.017 | 0.000 | **0.000** | 0.000 | **0.000** |
|  | **227** | 0.000 | 0.000 | 0.016 | 0.000 | 0.000 | **0.026** | **0.000** | 0.000 |
|  | **230** | 0.000 | 0.017 | 0.016 | 0.050 | 0.000 | 0.026 | **0.000** | 0.017 |
|  | **233** | 0.000 | 0.000 | 0.000 | 0.000 | 0.000 | 0.013 | 0.000 | 0.000 |
|  | **236** | 0.000 | 0.050 | 0.031 | 0.000 | 0.000 | 0.038 | 0.013 | **0.017** |
|  | **239** | 0.000 | 0.000 | 0.016 | 0.017 | **0.016** | **0.026** | 0.013 | 0.017 |
|  | **242** | 0.000 | 0.000 | 0.047 | 0.017 | **0.016** | **0.013** | 0.000 | 0.086 |
|  | **245** | 0.000 | 0.017 | 0.156 | 0.050 | 0.000 | 0.026 | 0.038 | 0.069 |
|  | **248** | 0.032 | 0.050 | 0.203 | 0.100 | 0.081 | 0.115 | 0.063 | 0.034 |
|  | **251** | 0.129 | 0.167 | 0.109 | 0.100 | 0.081 | 0.192 | 0.113 | 0.103 |
|  | **254** | 0.161 | 0.117 | 0.172 | 0.200 | 0.161 | 0.090 | 0.063 | 0.172 |
|  | **257** | 0.161 | 0.150 | 0.063 | 0.050 | 0.145 | 0.115 | 0.050 | 0.103 |
|  | **260** | 0.194 | 0.067 | 0.094 | 0.133 | 0.194 | 0.077 | 0.188 | 0.155 |
|  | **263** | 0.161 | 0.033 | 0.031 | 0.133 | 0.177 | 0.038 | 0.100 | 0.069 |
|  | **266** | 0.000 | 0.050 | 0.000 | 0.067 | **0.016** | **0.000** | **0.013** | 0.034 |
|  | **269** | 0.016 | 0.000 | 0.016 | 0.000 | **0.000** | 0.000 | 0.038 | 0.052 |
|  | **272** | 0.000 | 0.000 | 0.000 | 0.017 | 0.000 | 0.000 | 0.000 | **0.000** |
|  | **281** | 0.000 | 0.000 | 0.000 | 0.000 | 0.000 | 0.000 | 0.000 | 0.017 |
| **EC11** | **196** | 0.000 | 0.000 | 0.016 | 0.000 | 0.000 | 0.000 | **0.000** | **0.017** |
| (-0.049/-0.021) | **199** | 0.000 | 0.000 | 0.000 | 0.000 | 0.000 | 0.000 | 0.013 | 0.000 |
|  | **202** | 0.000 | 0.000 | 0.000 | 0.000 | **0.015** | 0.000 | **0.025** | 0.000 |
|  | **205** | 0.000 | 0.000 | 0.000 | 0.000 | 0.000 | 0.000 | 0.000 | 0.017 |
|  | **223** | 0.000 | 0.000 | 0.000 | 0.000 | 0.000 | 0.000 | 0.013 | 0.000 |
|  | **226** | 0.000 | 0.000 | 0.016 | 0.000 | 0.000 | 0.000 | **0.000** | **0.017** |
|  | **229** | 0.043 | 0.031 | 0.109 | 0.065 | 0.106 | 0.179 | 0.013 | 0.052 |
|  | **232** | 0.543 | 0.797 | 0.734 | 0.887 | 0.636 | 0.564 | 0.925 | 0.862 |
|  | **235** | 0.413 | 0.094 | 0.125 | 0.016 | 0.242 | 0.167 | 0.013 | 0.017 |
|  | **238** | 0.000 | 0.000 | 0.000 | 0.016 | 0.000 | **0.013** | 0.000 | 0.017 |
|  | **241** | 0.000 | 0.047 | 0.000 | 0.000 | 0.000 | 0.077 | 0.000 | 0.000 |
|  | **250** | 0.000 | 0.000 | 0.000 | 0.016 | 0.000 | 0.000 | 0.000 | **0.000** |
|  | **259** | 0.000 | 0.031 | 0.000 | 0.000 | 0.000 | **0.000** | 0.000 | 0.000 |
| **EC12** | **160** | 0.000 | 0.000 | 0.000 | 0.017 | 0.000 | 0.000 | 0.000 | 0.018 |
| (-0.101/-0.011) | **166** | 0.000 | 0.016 | 0.000 | 0.000 | 0.000 | **0.000** | 0.000 | 0.000 |
|  | **169** | 0.000 | 0.016 | 0.000 | 0.000 | 0.000 | 0.026 | 0.000 | **0.018** |
|  | **172** | 0.000 | 0.016 | 0.000 | 0.000 | **0.045** | 0.013 | 0.000 | 0.000 |
|  | **175** | 0.000 | 0.031 | 0.016 | 0.000 | **0.045** | **0.000** | **0.000** | 0.000 |
|  | **178** | 0.000 | 0.000 | 0.000 | 0.017 | 0.000 | 0.000 | 0.000 | 0.018 |
|  | **181** | 0.000 | 0.016 | 0.016 | 0.017 | **0.015** | **0.000** | 0.013 | 0.000 |
|  | **184** | 0.023 | 0.047 | 0.097 | 0.083 | 0.015 | 0.092 | 0.025 | 0.179 |
|  | **187** | 0.023 | 0.094 | 0.129 | 0.083 | 0.076 | 0.039 | 0.100 | 0.107 |
|  | **190** | 0.591 | 0.234 | 0.242 | 0.150 | 0.576 | 0.224 | 0.038 | 0.250 |
|  | **193** | 0.136 | 0.156 | 0.258 | 0.317 | 0.167 | 0.368 | 0.288 | 0.125 |
|  | **196** | 0.227 | 0.250 | 0.145 | 0.217 | 0.045 | 0.105 | 0.325 | 0.107 |
|  | **199** | 0.000 | 0.047 | 0.065 | 0.050 | 0.000 | 0.105 | 0.125 | 0.125 |
|  | **202** | 0.000 | 0.078 | 0.032 | 0.033 | **0.015** | 0.013 | 0.075 | 0.054 |
|  | **205** | 0.000 | 0.000 | 0.000 | 0.000 | 0.000 | **0.013** | **0.013** | 0.000 |
|  | **226** | 0.000 | 0.000 | 0.000 | 0.017 | 0.000 | 0.000 | 0.000 | 0.000 |
| **Eradic5** | **269** | 0.391 | 0.177 | 0.578 | 0.362 | 0.394 | 0.333 | 0.225 | 0.500 |
| (-0.017/0.049) | **272** | 0.000 | 0.000 | 0.000 | 0.000 | 0.000 | 0.000 | 0.025 | 0.000 |
|  | **275** | 0.609 | 0.823 | 0.422 | 0.638 | 0.606 | 0.667 | 0.750 | 0.500 |
| **Eradic10** | **448** | 0.000 | 0.000 | 0.000 | 0.000 | 0.000 | 0.000 | 0.013 | 0.000 |
| (0.036/0.096) | **451** | 0.016 | 0.065 | 0.016 | 0.000 | 0.015 | 0.064 | 0.088 | **0.067** |
|  | **454** | 0.078 | 0.048 | 0.016 | 0.000 | 0.015 | 0.026 | 0.075 | **0.033** |
|  | **457** | 0.906 | 0.887 | **0.250** | 0.200 | 0.955 | 0.910 | **0.775** | 0.133 |
|  | **460** | 0.000 | 0.000 | **0.625** | 0.700 | **0.015** | 0.000 | **0.050** | 0.650 |
|  | **463** | 0.000 | 0.000 | 0.078 | 0.050 | 0.000 | 0.000 | **0.000** | 0.050 |
|  | **466** | 0.000 | 0.000 | 0.000 | 0.050 | 0.000 | 0.000 | 0.000 | 0.067 |
|  | **469** | 0.000 | 0.000 | 0.016 | 0.000 | 0.000 | 0.000 | 0.000 | 0.000 |

**Figure S1.** Allele loadings for the first linear discriminant (LD1) of the DAPC plots (Fig. 3). Loadings are referring to coefficients of the alleles used in the linear combination. The continuous lines represent an arbitrary threshold of 0.07 under which alleles were not labelled and considered as not greatly contributing to the observed pattern.


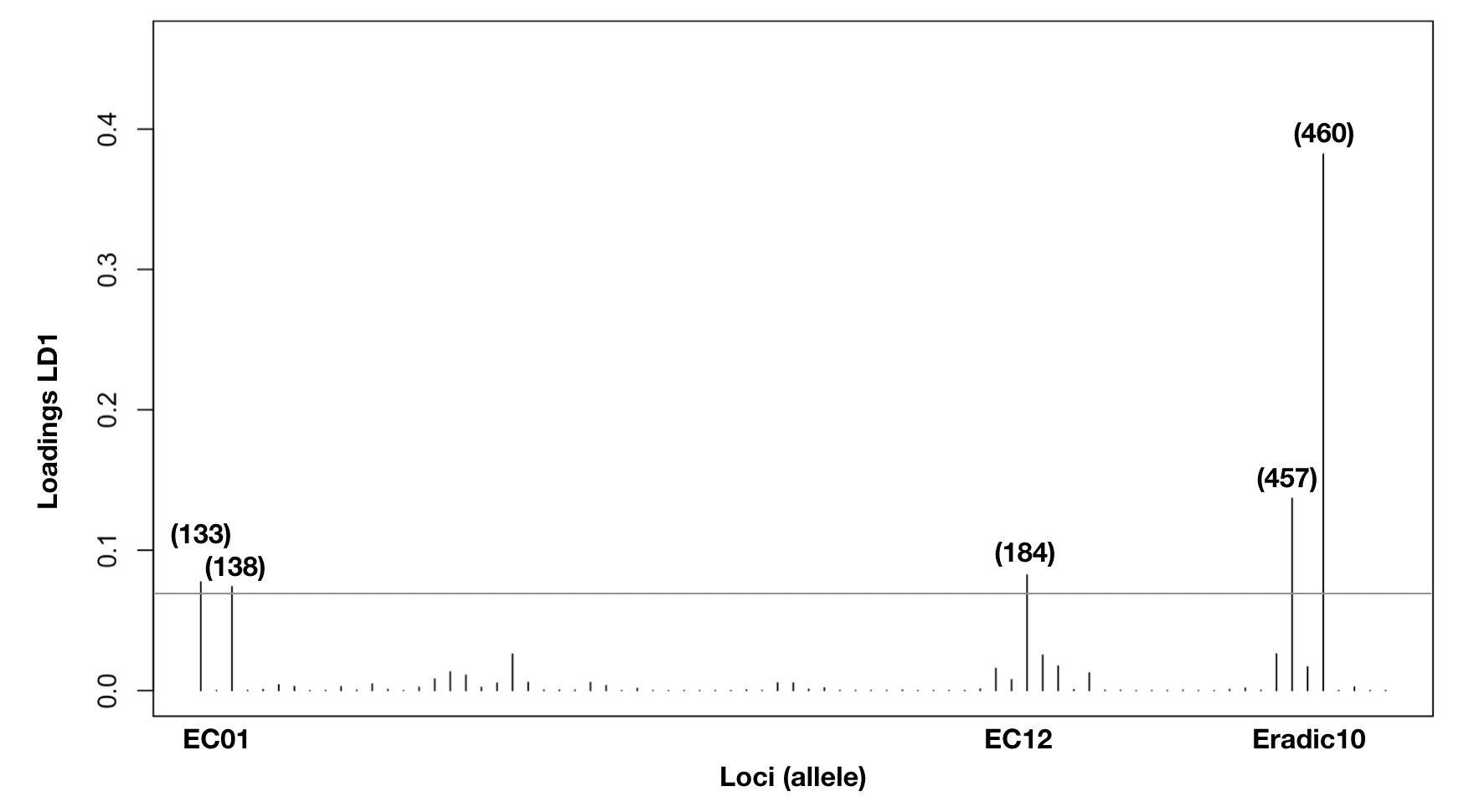

Supplement: Supplementary file 1 — Supplementary file1 [file 41598_2020_69665_MOESM1_ESM.docx]
